# LDL-C/HDL-C is associated with ischaemic stroke in patients with non-valvular atrial fibrillation: a case-control study

**DOI:** 10.1186/s12944-020-01392-7

**Published:** 2020-10-07

**Authors:** Xiao-Xue Zhang, Meng Wei, Lu-Xiang Shang, Yan-Mei Lu, Ling Zhang, Yao-Dong Li, Jiang-Hua Zhang, Qiang Xing, Zu Kela Tu-Erhong, Bao-Peng Tang, Xian-Hui Zhou

**Affiliations:** 1 Department of Cardiac Pacing and Electrophysiology, The First Affiliated Hospital of Xinjiang Medical University, No. 137, Liyushan Road, Urumqi, 830054 P R China; 2 Xinjiang Key Laboratory of Cardiac Electrophysiology and Remodeling, The First Affiliated Hospital of Xinjiang Medical University, Urumqi, 830054 P R China

**Keywords:** Non-valvular atrial fibrillation, Ischaemic stroke, Low−/high-density lipoprotein cholesterol ratio, Case-control study, Principal component regression analysis, Lipoproteins, Xinjiang

## Abstract

**Background:**

This study explored the relationships between the low−/high-density lipoprotein cholesterol ratio (LDL-C/HDL-C) and other clinical indicators and ischaemic stroke (IS) in patients with non-valvular atrial fibrillation (NVAF) in Xinjiang. The findings could provide a theoretical and therapeutic basis for NVAF patients.

**Methods:**

NVAF patients who were admitted to 10 medical centres across Xinjiang were divided into stroke (798 patients) and control (2671 patients) groups according to the occurrence of first acute IS. Univariate and multivariate logistic regression analysis were used to examine the independent risk factors for IS in NVAF patients. Factor analysis and principal component regression analysis were used to analyse the main factors influencing IS. Receiver operating characteristic (ROC) curve analysis was used to evaluate the discriminatory ability of LDL-C/HDL-C for predicting the occurrence of IS.

**Results:**

The stroke group had an average age of 71.64 ± 9.96 years and included 305 females (38.22%). The control group had a mean age of 67.30 ± 12.01 years and included 825 females (30.89%). Multivariate logistic regression showed that the risk of IS in the highest LDL-C/HDL-C quartile (≥2.73) was 16.23-fold that of the lowest quartile (< 1.22); IS risk was 2.27-fold higher in obese patients than in normal-weight subjects; IS risk was 3.15-fold higher in smoking patients than in non-smoking patients. The area under the ROC curve of LDL-C/HDL-C was 0.76, the optimal critical value was 2.33, the sensitivity was 63.53%, and the specificity was 76.34%. Principal component regression analysis showed that LDL-C/HDL-C, age, smoking, drinking, LDL-C and hypertension were risk factors for IS in NVAF patients.

**Conclusions:**

LDL-C/HDL-C > 1.22, smoking, BMI ≥24 kg/m^2^ and CHA_2_DS_2_-VASc score were independent risk factors for IS in NVAF patients; LDL-C/HDL-C was the main risk factor.

## Introduction

Atrial fibrillation (AF) is one of the most common arrhythmias, and it confers a four- to five-fold increase in the risk of ischaemic stroke (IS) compared with the absence of AF. In addition, IS caused by AF is particularly characterized by a mortality rate of 20% and a disability rate of 60% [1]. Compared with strokes not related to AF, AF-related strokes have high rates of disability and a mortality rate that is twice as high as that of non-AF-related stroke [2]. With or without anticoagulant therapy, Asian patients with AF were more likely to have IS than non-Asian patients; furthermore, they had a higher risk of haemorrhagic stroke [1, 3, 4]. The incidence rate of AF increases sharply with age, and ageing is one of the most significant hazard factors for AF [5, 6]. The population of Asia is ageing rapidly. It is estimated that in 2050, there will be approximately 72 million patients with AF, of whom 2.9 million may have AF-related stroke [7]. The management costs of AF are high—approximately 10,100–14,200 dollars per person in the United States and 450–3000 dollars per person in Europe [8]. Therefore, the prevention of IS caused by AF is an important part of the treatment strategy for AF.

CHADS_2_ and CHA_2_DS_2_-VASc scores are currently widely used to appraise the risk stratification of stroke and thromboembolism in AF patients [9]. Oral anticoagulants (OACs) are recommended for patients at high risk of stroke [9]. CHADS_2_ and CHA_2_DS_2_-VASc scores are practical tools for evaluating the prognosis of stroke patients [10]. However, these above-mentioned two scoring systems do not take into account other potential risk factors, such as renal function impairment, rheumatoid arthritis, obesity or smoking, nor do they consider clinically accessible laboratory indicators, imaging values and other parameters [11, 12]. Friberg et al. [13] have shown that clinically diagnosed heart failure does not increase rates of stroke in AF patients. Other studies have shown that left atrial enlargement and low-density lipoprotein cholesterol (LDL-C) are clinically easy-to-obtain indicators for risk stratification of thromboembolism events in patients with AF [14–16]. Additionally, high-density lipoprotein cholesterol (HDL-C) has been shown to be negatively correlated with the risk or severity of IS [17, 18]. The LDL-C/HDL-C ratio has also been shown to have greater predictive value of risk in cardiovascular disease than either of its individual components, particularly LDL-C [19]. However, data on the association between LDL-C/HDL-C and IS in AF patients are limited, and the aetiology of IS is complex. Therefore, the present study intends to investigate the relationships between LDL-C/HDL-C and other clinical indicators and IS in non-valvular atrial fibrillation (NVAF) patients in Xinjiang to provide a basis for the prevention, treatment and the comprehensive management of patients with AF.

## Methods

### Study design and participants

This study was approved by the ethics committee of First Affiliated Hospital of Xinjiang Medical University (Ethics Approval Number: 20140925–04). All participants signed written informed consent.

This was a retrospective case-control study that consecutively enrolled all patients with NVAF who were admitted with their first acute IS at 10 comprehensive large hospitals in different regions of Xinjiang between January 1st, 2017 and January 1st, 2019. Patients with NVAF who were admitted to the same hospital at the same time for any reason other than acute IS were selected as controls. The inclusion criteria were as follows: (1) patients with NVAF ≥18 years old and (2) acute IS confirmed by brain magnetic resonance imaging (MRI) or computed tomography (CT). According to a cerebrovascular aetiology analysis, the cause of stroke was cardiogenic embolism. The exclusion criteria were as follows: (1) congenital heart valve disease, rheumatic heart valve disease, heart valve replacement, senile heart valve disease and other heart valve diseases; (2) malignant tumour, chronic kidney or liver diseases, and thyroid diseases; (3) ischaemic hypoxic encephalopathy, dementia and other intracranial lesions; (4) aetiology involving systemic inflammatory reaction, acute myocardial infarction and other reversible factors; (5) AF diagnosis after IS; and (6) patients with incomplete medical records who had missing necessary data.

### AF and IS assessments

The diagnosis of AF was obtained from 12-lead electrocardiographs (ECG) or 24-Holter ECG monitor recordings [9] or a history of AF diagnosed by a cardiologist. IS was diagnosed by the diagnostic criteria: an episode of acute onset; focal neurological deficit caused by focal cerebral infarction; responsible ischaemic brain lesions observed with CT or MRI; and no evidence of frank blood on brain CT or MRI [20]. Different CT and MRI models were used in all hospitals. They were of the same quality and sensitivity in IS diagnosis.

### Data collection and lipid profile analyses

The following baseline data were recorded: sex, age, smoking status, body mass index (BMI), comorbidities (hypertension, diabetes mellitus, heart failure, and vascular disease) and medication history. The CHA_2_DS_2_-VASc score (one point are given for heart failure, hypertension, diabetes mellitus, age 65–74 years, vascular disease and female sex; two points each for age over 75 years and previous stroke/transient ischaemic attack [TIA]/thromboembolism) was used to appraise the risk of IS in AF patients. Fasting venous blood samples were obtained from all subjects on the second day of hospitalization. Concentrations of blood lipids (HDL-C, total cholesterol [TC], triglycerides [TG] and LDL-C) and other indicators (fasting plasma glucose, blood urea nitrogen, creatinine and uric acid) were directly measured with an automatic blood cell analyser. Non-HDL-C = TC-HDL-C.

### Statistical analysis

The data were analysed by SPSS version 21.0 (SPSS, Inc., Chicago, IL, USA) and MedCalc (MedCalc Software, Mariakerke, Belgium). Continuous data with a normal distribution were expressed as the mean ± standard deviation (SD), and comparisons between groups were performed with two independent samples Student’s t tests. Non-normal continuous variables were presented as median (inter-quartile range [IQR]), and intergroup comparisons were performed with the Mann-Whitney U test. Data were presented as numbers and percentages for categorical variables, and were compared by the chi-square test. Univariate logistic regression analysis was used to examine the independent risk factors for IS in NVAF patients. Variables that were significant in the univariate logistic regression analysis (CHA_2_DS_2_-VASc score, smoking, LDL-C/HDL-C quartiles, BMI) were included in the multivariate logistic regression analysis. Multivariable adjusted analysis was performed using three models based on the LDL-C/HDL-C quartile stratification. The models were as follows: model 1 adjusted for age and sex; model 2 adjusted for CHA_2_DS_2_-VASc score; and model 3 adjusted for uric acid, creatinine, blood urea nitrogen, fasting blood glucose, TG, TC, non-HDL-C, smoking, drinking, and BMI. Factor analysis and principal component regression analysis were used to analyse the main influencing factors of IS. Receiver operating characteristic (ROC) curve analysis and calibration plot were used to evaluate the discriminatory and calibration ability of the LDL-C/HDL-C before and after the inclusion of CHA_2_DS_2_-VASc score. The pairwise comparison of ROC curves (using the De Long method) was performed using Z statistics. *P* < 0.05 was considered statistically significant.

## Results

### Baseline characteristics

A total of 798 cases and 2671 controls were recruited. The average age was 68.30 ± 11.72 years. The age, BMI, CHA_2_DS_2_-VASc score, LDL-C/HDL-C and LDL-C levels of the stroke group were higher than those of the control group (all *P* < 0.05). The HDL-C level of the stroke group was lower than that of the control group (*P* < 0.01). The proportions of female sex, smoking and hypertension in the stroke group were higher than those in the control group (all *P* < 0.05) (Table 1).

**Table 1:** Baseline characteristics of non-valvular atrial fibrillation patients with and without ischaemic stroke

Demographic characteristics	Stroke		t/χ^2^/Z	*P*
	Yes (*n* = 798)	No (*n* = 2671)		
Gender				
Women, n (%)	305 (38.22)	825 (30.89)	15.04	< 0.01
Men, n (%)	493 (61.78)	1846 (69.11)		
Age, year	71.64 ± 9.96	67.30 ± 12.01	−10.28	< 0.01
Body mass index, kg/m^2^	25.20 ± 3.26	24.75 ± 3.35	−3.35	< 0.01
Hypertension, n (%)	352 (44.11)	907 (33.96)	27.39	< 0.01
Diabetes mellitus,n (%)	171 (21.43)	531 (19.88)	0.91	0.34
Heart failure, n (%)	424 (53.13)	1510 (56.53)	2.88	0.09
Vascular disease, n (%)	121 (15.16)	263 (9.85)	17.64	< 0.01
CHA_2_DS_2_-VASc score, mean	4.96 ± 1.37	2.47 ± 1.40	−44.45	< 0.01
Smoking, n (%)	269 (33.71)	660 (24.71)	25.38	< 0.01
Drinking, n (%)	124 (15.54)	371 (13.89)	1.37	0.24
TG, mmol/L	1.11 ± 0.67	1.14 ± 0.89	1.08	0.28
TC, mmol/L	3.57 ± 1.29	3.48 ± 1.47	−1.55	0.12
non-HDL-C, mmol/L	2.65 ± 1.25	2.48 ± 1.42	−3.10	< 0.01
LDL-C, mmol/L	2.58 ± 1.02	1.69 ± 0.86	−22.18	< 0.01
HDL-C, mmol/L	0.92 ± 0.39	1.00 ± 0.34	5.47	< 0.01
LDL-C/HDL-C	2.83 (1.83, 4.09)	1.55 (1.10, 2.27)	−22.17	< 0.01
Uric acid, umol/L	278.54 ± 147.06	295.71 ± 145.64	2.92	< 0.01
Creatinine, mmol/L	75.44 ± 37.23	76.00 ± 59.92	0.32	0.75
Blood urea nitrogen, mmol/L	5.97 ± 2.68	6.05 ± 2.77	0.67	0.50
Fasting plasma glucose, mmol/L	5.93 ± 2.44	5.75 ± 2.29	−1.92	0.06
Previous Medications				
Anti-hypertensive drugs, n (%)	287 (35.96)	754 (28.23)	17.51	< 0.01
Anti-diabetic agents, n (%)	141 (17.67)	420 (15.72)	1.71	0.19
Statins, n (%)	85 (10.65)	345 (12.92)	2.90	0.09
anti-platelet drugs, n (%)	150 (18.80)	583 (21.83)	3.39	0.07
anticoagulant drugs, n (%)	189 (23.68)	656 (24.56)	0.26	0.61

*HDL-C* high-density lipoprotein cholesterol, *LDL-C* low-density lipoprotein cholesterol, *non-HDL-C* non-high-density lipoprotein cholesterol, *TC* total cholesterol, *TG* triglycerides

### Univariate logistic regression analysis of IS

Univariate logistic regression showed that the highest quartile (fourth quartile, ≥2.73) of LDL-C/HDL-C had an OR of 13.26 (95% CI: 9.84–17.86, *P* < 0.01) compared with the bottom quartile (first quartile, < 1.22) (Table 2). LDL-C levels (OR: 5.57, 95% CI: 3.53–8.80, *P* < 0.01) were also found to be a risk factor for IS in patients with NVAF. High levels of the HDL-C had an OR of 0.81 (95% CI: 0.70–0.95, *P* = 0.01) compared with the low levels. Other significant correlates of IS were age, CHA_2_DS_2_-VASc score, smoking and BMI (all *P* < 0.05).

**Table 2:** Univariate logistic regression analysis for risk factors of ischaemic stroke

Characteristic	β	SE	Waldχ^2^	OR	95% CI	*P*
Age (years)						
< 65	Reference					
65 to 74	0.57	0.11	27.74	1.77	1.43, 2.20	< 0.01
≥ 75	0.89	0.11	72.50	2.44	1.99, 3.00	< 0.01
Women	0.33	0.08	14.98	1.38	1.17, 1.63	< 0.01
Hypertension	0.43	0.08	27.19	1.54	1.31, 1.80	< 0.01
Diabetes mellitus	0.10	0.10	0.91	1.10	0.91, 1.33	0.34
Vascular disease	0.49	0.12	17.38	1.64	1.30, 2.06	< 0.01
CHA_2_DS_2_-VASc score	1.27	0.05	724.32	3.55	3.24, 3.90	< 0.01
Smoking	0.44	0.09	25.16	1.55	1.31, 1.84	< 0.01
Drinking	0.13	0.11	1.36	1.14	0.92, 1.42	0.24
Heart failure	−0.14	0.08	2.88	0.87	0.74, 1.02	0.09
TG (mmol/L)						
≤ 2.26	Reference					
> 2.26	−0.19	0.21	0.77	0.83	0.55, 1.26	0.38
TC (mmol/L)						
≤ 6.22	Reference					
> 6.22	0.16	0.31	0.26	1.18	0.64, 2.17	0.61
non-HDL-C (mmol/L)	0.09	0.03	9.03	1.09	1.03, 1.16	< 0.01
LDL-C (mmol/L)						
≤ 4.14	Reference					
> 4.14	1.72	0.23	54.25	5.57	3.53, 8.80	< 0.01
HDL-C (mmol/L)						
≥ 1.04	−2.05	0.08	6.47	0.81	0.70, 0.95	0.01
< 1.04	Reference					
LDL-C/HDL-C						
First quartile (< 1.22)	Reference					
Second quartile [1.22, 1.71)	0.69	0.17	16.48	2.00	1.43, 2.79	< 0.01
Third quartile [1.71, 2.73)	1.48	0.16	88.09	4.39	3.22, 5.79	< 0.01
Fourth quartile (≥2.73)	2.58	0.15	289.19	13.26	9.84, 17.86	< 0.01
Body mass index (kg/m^2^)						
< 24	Reference					
[24, 28)	0.43	0.09	23.18	1.54	1.29, 1.83	< 0.01
≥ 28	0.34	0.12	7.77	1.41	1.11, 1.79	< 0.01
Uric acid (umol/L)	−0.001	0.00	8.46	1.00	0.999, 1.000	< 0.01
Creatinine (mmol/L)	0.00	0.001	0.06	1.00	0.998, 1.001	0.80
Blood urea nitrogen (mmol/L)	−0.01	0.02	0.45	0.99	0.96, 1.02	0.50
Fasting plasma glucose (mmol/L)						
< 7	Reference					
≥ 7	0.13	0.10	1.62	1.13	0.94, 1.38	0.20

*HDL-C* high-density lipoprotein cholesterol, *LDL-C* low-density lipoprotein cholesterol, *non-HDL-C* non-high-density lipoprotein cholesterol, *TC* total cholesterol, *TG* triglycerides, *95% CI* 95% confidence interval, *OR* odds ratio, *SE* standard error

### Multivariate logistic regression analysis of IS

Multivariate logistic regression showed that the risk of IS in the highest quartile of LDL-C/HDL-C (≥ 2.73) was 16.23-fold higher than that in the lowest quartile (< 1.22) (Table 3). The risk of IS was 2.27-fold higher in obese patients (BMI ≥ 28 kg/m^2^) than in normal-weight subjects. LDL-C/HDL-C, BMI, smoking and CHA_2_DS_2_-VASc score were independent risk factors for IS in NVAF patients. Since LDL-C/HDL-C provides comprehensive blood lipid information, a multivariate calibration was then conducted. The results showed that the multivariable-adjusted ORs of IS in different regression models increased linearly (*P* < 0.01) (Table 4). Compared with the lowest quartile, the risk of IS in the highest quartile of LDL-C/HDL-C was still significantly increased after adjusting for CHA_2_DS_2_-VASc score and other confounding variables.

**Table 3:** Multivariate logistic regression analysis for risk factors of ischaemic stroke

Characteristic	β	SE	Waldχ^2^	OR	95% CI	*P*
CHA_2_DS_2_-VASc score	1.41	0.06	635.68	4.08	3.66, 4.56	< 0.01
Smoking	1.15	0.14	69.59	3.15	2.41, 4.13	< 0.01
LDL-C/HDL-C						
First quartile (< 1.22)	Reference					
Second quartile [1.22, 1.71)	0.80	0.21	14.50	2.22	1.47, 3.36	< 0.01
Third quartile [1.71, 2.73)	1.65	0.20	68.16	5.21	3.52, 7.72	< 0.01
Fourth quartile (≥2.73)	2.79	0.20	194.65	16.23	10.97, 24.01	< 0.01
Body mass index, kg/m^2^						
< 24	Reference					
[24, 28)	0.71	0.14	27.40	2.03	1.56, 2.65	< 0.01
≥ 28	0.82	0.19	18.80	2.27	1.57, 3.29	< 0.01

*HDL-C* high-density lipoprotein cholesterol, *LDL-C* low-density lipoprotein cholesterol, *95% CI* 95% confidence interval, *OR* odds ratio, *SE* standard error

**Table 4:** Multivariable adjusted odds ratios of LDL-C/HDL-C quartiles in relation to risk factor of ischaemic stroke

Variables	LDL-C/HDL-C quartiles				*P*
	Q1 (*n* = 865)	Q2 (*n* = 869)	Q3 (*n* = 868)	Q4 (*n* = 867)	
ischaemic stroke case, n (%)	58 (7.27)	109 (13.66)	208 (26.07)	423 (53.00)	< 0.01
Range of LDL-C/HDL-C quartile	< 1.22	[1.22, 1.71)	[1.71, 2.73)	≥2.73	
Model1	1.00	2.14 (1.53, 2.99)	5.11 (3.73, 6.99)	16.00 (11.79, 21.71)	< 0.01
Model2	1.00	2.37 (1.60, 3.53)	6.05 (4.13, 8.84)	17.34 (11.87, 25.32)	< 0.01
Model3	1.00	2.56 (1.82, 3.62)	6.19 (4.46, 8.60)	24.45 (17.18, 34.79)	< 0.01

Model1 adjusted for age and sex; Model2 adjusted for CHA_2_DS_2_-VASc score; Model3 adjusted for blood urea nitrogen, BMI, creatinine, drinking, fasting plasma glucose, non-HDL-C, smoking, triglyceride, total cholesterol, and uric acid. *Abbreviations*: *BMI* body mass index, *HDL-C* high-density lipoprotein cholesterol, *LDL-C* low-density lipoprotein cholesterol, *non-HDL-C* non-high-density lipoprotein cholesterol

### LDL-C/HDL-C as a predictor of IS

**Fig. 1:**
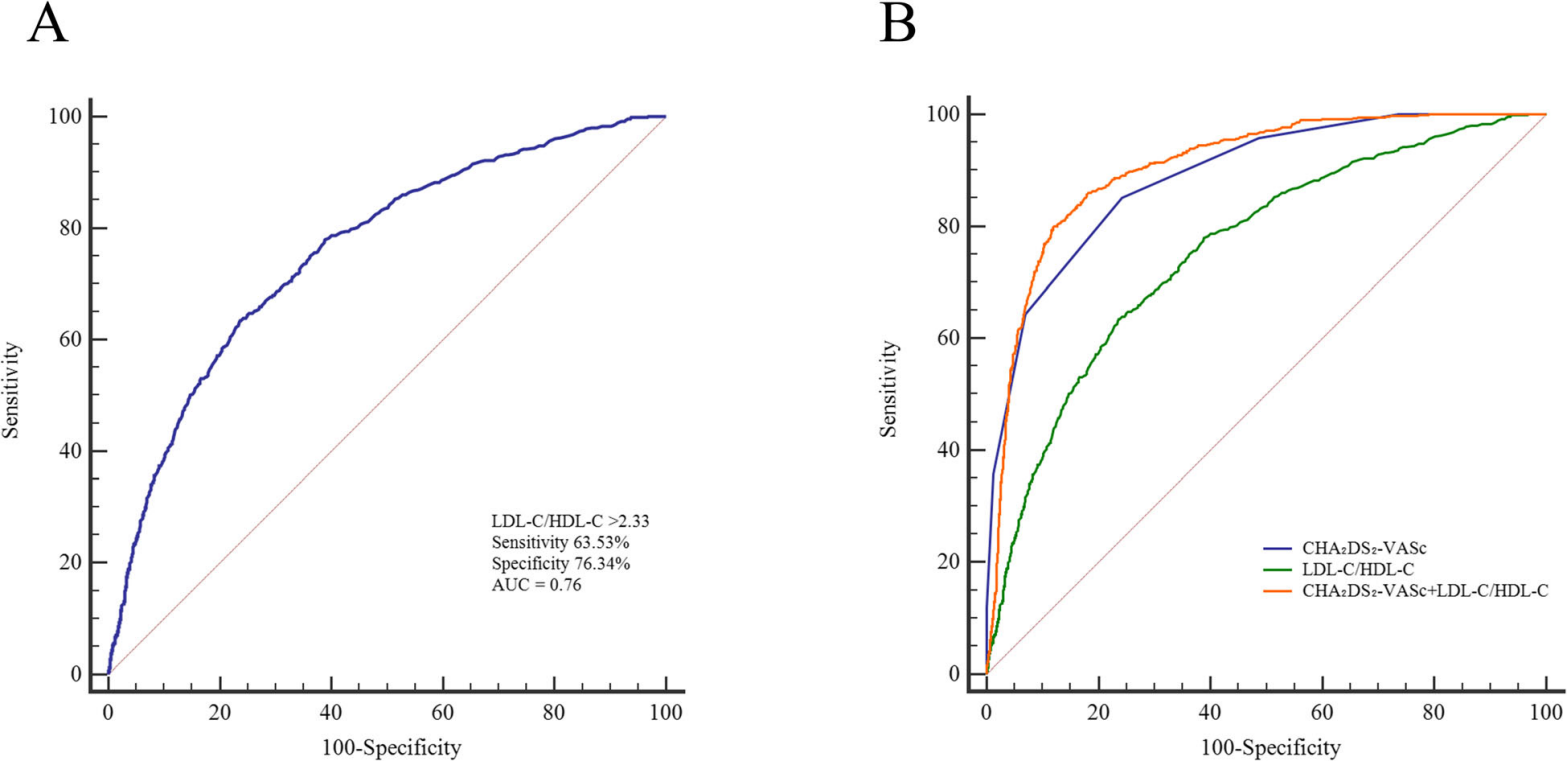
**a** The ROC curve of LDL-C/HDL-C to predict ischaemic stroke in patients with non-valvular atrial fibrillation; **b** The ROC curves for the CHA_2_DS_2_-VASc score and the combination of CHA_2_DS_2_-VASc score and LDL-C/HDL-C. Abbreviations: HDL-C = high-density lipoprotein cholesterol; LDL-C = low-density lipoprotein cholesterol

**Fig. 2:**
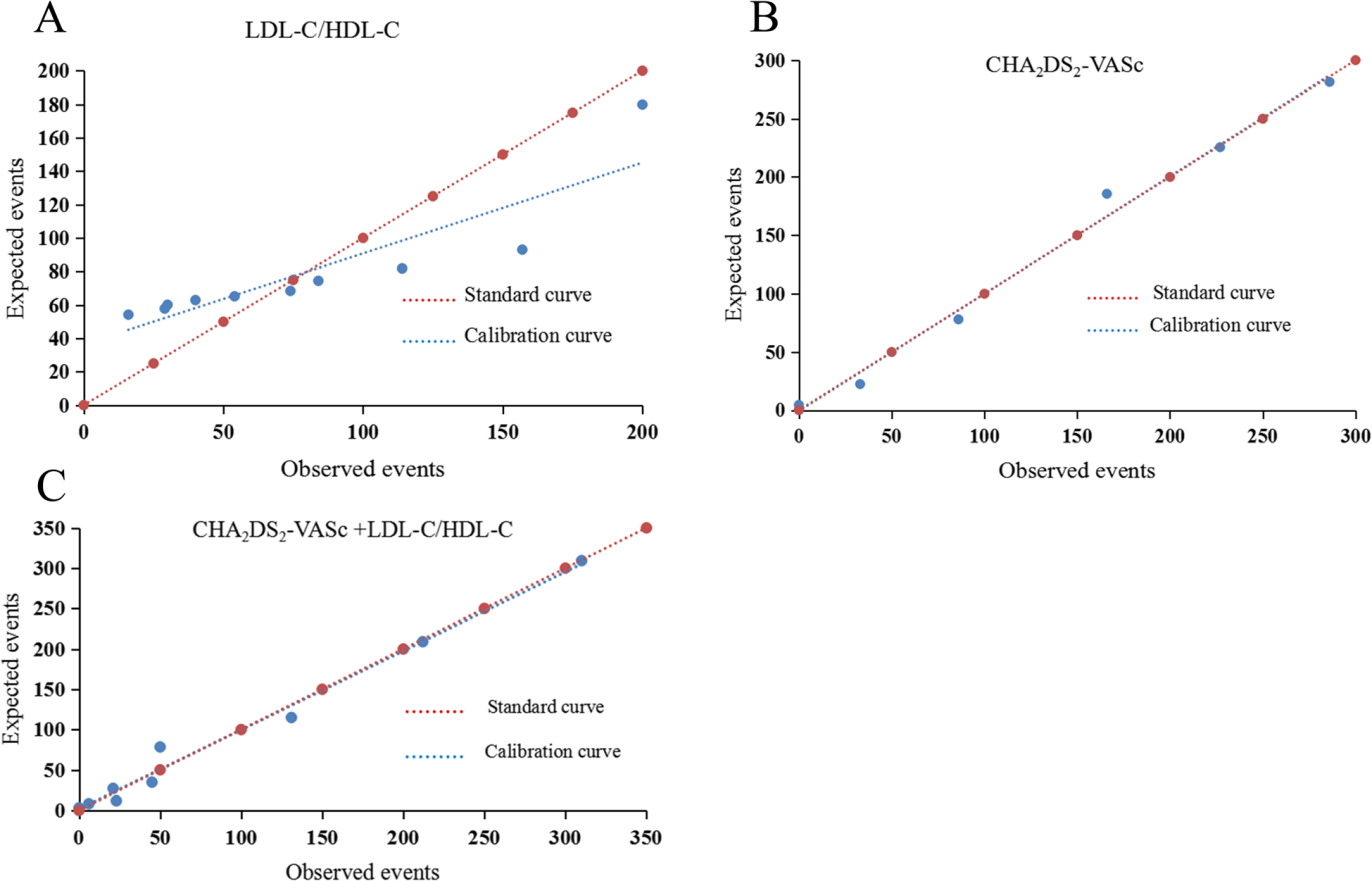
**a** The calibration plot of LDL-C/HDL-C; **b** The calibration plot of for the CHA_2_DS_2_-VASc score; **c** The calibration plot of LDL-C/HDL-C plus CHA_2_DS_2_-VASc score. Abbreviations: HDL-C = high-density lipoprotein cholesterol; LDL-C = low-density lipoprotein cholesterol

The area under the ROC curve (AUC) of LDL-C/HDL-C was 0.76, the optimal critical value was 2.33, the sensitivity was 63.53%, and the specificity was 76.34% (Table 5). The AUC of the CHA_2_DS_2_-VASc score was 0.89, the sensitivity was 85.09%, and the specificity was 75.78%. Additionally, the AUC of the CHA_2_DS_2_-VASc score plus LDL-C/HDL-C was higher than that of the CHA_2_DS_2_-VASc score alone (0.91 vs. 0.89, Z = 3.26, *P* < 0.01). These results suggested that the power of the CHA_2_DS_2_-VASc score for predicting the risk of stroke in patients with NVAF was improved after the addition of LDL-C/HDL-C (Fig. 1). The calibration curve and the standard curve of LDL-C/HDL-C plus CHA_2_DS_2_-VASc score almost completely coincided, indicating that this model had good calibration and high accuracy for predicting the risk of IS (Fig. 2).

**Table 5:** Areas under the receiver operating characteristic curve (AUC) of LDL-C/HDL-C, CHA_2_DS_2_-VASc and LDL-C/HDL-C + CHA_2_DS_2_-VASc

Variables	Sensitivity	Specifificity	Cut-off value	AUC	SE	95% CI	*P*
LDL-C/HDL-C	63.53	76.34	2.33	0.76	0.01	0.74, 0.77	< 0.01
CHA_2_DS_2_-VASc	85.09	75.78	3.00	0.89	0.01	0.88, 0.90	< 0.01
LDL-C/HDL-C + CHA_2_DS_2_-VASc	79.95	87.98	6.26	0.91	0.01	0.90, 0.92	< 0.01

*HDL-C* high-density lipoprotein cholesterol, *LDL-C* low-density lipoprotein cholesterol, *95% CI* 95% confidence interval, *SE* standard error

### Factor analysis and IS influencing factors

Factor analysis was performed for ten clinical indexes. The Kaiser-Meyer-Olkin (KMO) test value was 0.53, and the Bartlett test showed that χ^2^ = 557.85, *P* < 0.01. The results of the two tests showed a strong correlation between indicators, and the data were suitable for factor analysis. As Table 6 shows, the variable correlation matrix contains the five largest eigenvalues, namely, 1.73, 1.43, 1.30, 1.25, and 1.08, and the cumulative contribution rate of variance is 67.85%, which shows that the first five principal components provide most of the information contained in the original data. Principal component 1 (PC1) mainly represents LDL-C/HDL-C and HDL-C, PC2 mainly represents smoking and drinking, PC3 mainly represents TC and TG, PC4 mainly represents LDL-C and age, and PC5 mainly represents hypertension. The factor names are as follows: PC1 is the blood lipid ratio factor, PC2 is the bad living habits factor, PC3 is the blood lipid-related factor, PC4 is the age-related factor, and PC5 is the blood pressure-related factor. Multivariate unconditional logistic regression analysis was performed with the five common factors (PC1-PC5) as covariates and with the absence or presence of IS as the dependent variable. The results showed that, with the exception of PC3, the other factors were risk factors for IS in patients with AF (Table 7). The standardized principal regression equation was as follows: Zy = 0.53 (PC1) + 0.11 (PC2) + 0.99 (PC4) + 0.23 (PC5) -1.45.

**Table 6:** Factor analysis: loadings of related variables of ischaemic stroke in atrial fibrillation patients

Variables	PC1	PC2	PC3	PC4	PC5
HDL-C	−0.91	− 0.01	0.10	0.21	0.05
LDL-C/HDL-C	0.89	− 0.03	0.05	0.27	−0.01
Drinking	−0.02	0.84	0.06	−0.05	0.08
Smoking	0.00	0.83	−0.09	− 0.02	− 0.06
TG	0.08	0.00	0.79	−0.06	−0.11
TC	−0.22	− 0.08	0.66	0.26	0.28
LDL-C	0.03	−0.01	0.21	0.86	−0.16
Age	0.02	−0.11	−0.38	0.56	0.33
Hypertension	0.18	−0.05	0.17	−0.12	0.82
Diabetes mellitus	0.16	−0.05	0.08	−0.06	−0.42
Eigenvalue	1.73	1.43	1.30	1.25	1.08
Explained variance (%)	17.27	14.25	13.04	12.49	10.78
Cumulative variance (%)	17.27	31.52	44.57	57.06	67.85

*HDL-C* high-density lipoprotein cholesterol, *LDL-C* low-density lipoprotein cholesterol, *PC* principal component, *TC* total cholesterol, *TG* triglycerides

**Table 7:** Principal component regression analysis

	β	S E	Waldχ^2^	OR	95% CI	*P*
PC1	0.53	0.05	107.44	1.70	1.54, 1.88	< 0.01
PC2	0.11	0.04	7.46	1.12	1.03, 1.22	< 0.01
PC3	0.04	0.04	0.86	1.04	0.96, 1.12	0.35
PC4	0.99	0.05	390.52	2.69	2.44, 2.96	< 0.01
PC5	0.23	0.05	21.83	1.26	1.15, 1.39	< 0.01
Constant	−1.45	0.05	882.44	0.24		< 0.01

*PC* principal component, *95% CI* 95% confidence interval, *OR* odds ratio, *SE* standard error

## Discussion

The most common complication of AF is thromboembolism, especially IS [6]. AF-related IS has high rates of mortality and disability. Because of the combination of high morbidity with a low diagnosis rate for cardiogenic stroke, it is of great significance to accurately identify high-risk patients and provide timely treatment to prevent the occurrence of IS in patients with AF. Currently, there is no risk model that can accurately predict IS in patients with AF. A large sample cohort study showed that the C-statistic of CHA_2_DS_2_-VASc score was 0.68, with only moderate predictive capacity [21]. Therefore, the present study explored the risk factors for IS in patients with NVAF in Xinjiang and provided the basis for further clinical treatment. First, multivariate logistic regression analysis showed that LDL-C/HDL-C > 1.22, smoking, BMI ≥ 24 kg/m^2^ and CHA_2_DS_2_-VASc score were independent risk factors for IS in patients with NVAF. Second, principal component regression analysis showed that LDL-C/HDL-C, age, smoking, drinking, hypertension and LDL-C were risk factors for IS in NVAF patients.

In this study, high LDL-C/HDL-C was found to be an independent risk factor for IS after adjusting for age and other related factors, indicating that high LDL-C/HDL-C may influence the progression of IS through particular pathways. The potential mechanism of the positive correlation between LDL-C/HDL-C and IS in NVAF patients remains unclear; however, there are several possible mechanisms that could explain this phenomenon. First, LDL-C/HDL-C indicates the proportions of atherosclerotic and anti-atherosclerotic lipoproteins, thus offering improved power for predicting the development of atherosclerosis. High LDL-C/HDL-C may indicate vulnerability to atherosclerotic plaques, which are prone to plaque rupture and thrombosis and eventually lead to IS. Okuzumi A et al. [22] indicated that high LDL-C/HDL-C was significantly correlated the vulnerability of aortic plaque in patients with IS. Second, LDL-C/HDL-C may be closely related to inflammation because HDL-C has anti-inflammatory and antioxidant properties [23], and LDL-C may be correlated with inflammation [24]. The high LDL-C/HDL-C ratio may be due to an increase in inflammatory components, a decrease in the anti-inflammatory and antioxidative components reflected in the denominator, or both. Pinto A et al. [25] observed that inflammatory markers, including TNF-α, IL-6 and von Willebrand factor (vWF), were predictors of new-onset IS in patients with chronic NVAF. Additionally, vWF has been shown to aggravate ischaemic injury by promoting thrombosis in the injured vessels, a finding that provides a new target for antithrombotic therapy [26, 27]. Inflammation has been confirmed to be associated with left atrial thrombosis in AF patients [28]. Previous studies have found that inflammatory biomarkers are significantly associated with left atrium or left atrial appendage thrombus outcomes in AF patients [29]. In conclusion, LDL-C/HDL-C may cause IS in NVAF patients by promoting atherosclerosis and left atrial or left atrial appendage thrombosis.

Studies have found that smoking affects serum lipid metabolism, increasing the level of LDL-C while decreasing HDL-C [30, 31]. After smoking cessation, LDL-C levels remain unchanged, while HDL-C levels increased [32]. Nicotine and oxygen free radicals in tobacco cause or aggravate vascular endothelial dysfunction, atherosclerosis and hypercoagulability through a variety of mechanisms [33–35]. These factors can promote thrombosis. Previous studies have shown that smoking increases the risk of thromboembolism or death in AF patients [36, 37]. Incorporating smoking as a risk factor for IS in CHADS_2_ and CHA_2_DS_2_-VASc scores could better predict the risk of IS in male patients [38]. Weight gain increases the LDL-C concentration and decreases the level of HDL-C [39, 40]. With increases in BMI, serum lipids and blood viscosity increase significantly, leading to thromboembolism and IS [41]. The present study found that an increase in BMI is an important risk factor for IS in NVAF patients. Previous studies have shown that BMI is negatively correlated with IS in AF patients [42], which is contrary to the results of the present study. At present, the relationship between obesity and IS remains controversial [42, 43]. Large sample, multicentre and prospective studies are needed to further explore the relationship between BMI and serum lipids and the influence of this relationship on adverse event outcomes in AF patients. In conclusion, smoking and BMI can increase the LDL/HDL ratio, which may lead to an increased risk of IS in NVAF patients by promoting atherosclerosis and cardiogenic thromboembolism.

Age is a risk factors for IS in AF patients. Previous studies have shown that AF is a disease of ageing, and with increasing age, the incidence of AF and stroke increases [5, 44]. With increasing age, LDL-C and HDL-C show an upward trend [45]. However, other researches reached different or opposing conclusions [46]. There is a close relationship between serum lipids and age, but conclusions differ among research studies, possibly due to age, geographical and ethnic differences in study populations. Moderate drinking can increase HDL-C and decrease LDL-C levels [47], which seems to be beneficial for reducing the risk of cardiovascular disease. However, the effects of drinking on IS are multifaceted and complex. Alcohol intake is a risk factor for thromboembolism, which may offset the protective effect of serum lipids through unknown mechanisms. Studies have found that long-term drinking can cause vascular haemodynamic changes, altered blood viscosity, and enhanced platelet aggregation, which subsequently promote the occurrence of IS [48]. The Stroke Prevention in Atrial Fibrillation (SPAF) I-III trials found that the incidence of IS in patients with AF who regularly drank a small amount of alcohol was lower than that in patients who did not drink alcohol [49]. In contrast, heavy drinking was related to a higher risk of IS [50]. Patients with hypertension have increased LDL-C levels [51] and normal or reduced levels of HDL-C [51, 52]. It has been shown that statins can reduce blood pressure when they are taken for lipid-lowering therapy [53], indicating that hypertension is closely related to dyslipidaemia. Additionally, hypertension is closely related to stroke, and active and effective control of blood pressure can reduce the incidence of IS [54]. Anti-hypertensive treatment can therefore reduce the incidence of stroke in hypertension patients [55]. In terms of mechanisms, hypertension can cause vascular haemodynamic changes, leading to atherosclerosis, and stenosis of the lumen, and can affect the blood supply of brain tissue [56]. At the same time, hypertension can promote the remodelling of left atrial structure and function and eventually lead to atrial fibrosis and electrical activity changes [57]. These changes, together with local or systemic inflammatory reactions, lead to local thrombosis or atherosclerotic thrombosis in the left atrium [57]. In conclusion, age, alcohol consumption, hypertension and serum lipid levels are closely related, and IS also closely related to these factors, which can lead to the occurrence of IS through the effects of lipoproteins and other mechanisms (as mentioned above).

The results show that the PC1 had the highest contribution (17.27%), and the LDL-C/HDL-C and HDL-C had the highest loads. HDL-C was negatively correlated with IS, and LDL-C/HDL-C was positively correlated with IS. The contribution rate of PC2 was 14.25%, and the factor loads of smoking and drinking were the highest, suggesting that bad living habits are among the risk factors for IS in AF patients. The contribution rate of PC4 was 12.49%. LDL-C and age had the highest factor loads, and they were positively correlated with IS. PC5 had the lowest contribution rate (10.78%), and hypertension was the factor with the highest load, suggesting that blood pressure is the factor that influences IS in AF patients. These results suggest that in clinical practice, in addition to the use of the classic CHA_2_DS_2_-VASc score to assess the risk of AF stroke, blood lipid-related parameters should be considered, as well as poor living habits and other factors. Blood lipid-related parameters include LDL-C/HDL-C, which comprehensively considers the impact of blood lipids on stroke and is a better indicator than either measure alone.

In summary, the findings of present study demonstrated that LDL-C/HDL-C, smoking, BMI, age, alcohol consumption, LDL-C and hypertension were risk factors for IS in NVAF patients. LDL-C/HDL-C is the main risk factor, which manifests that LDL-C/HDL-C may help identify AF individuals who are at high risk of IS and who may benefit from lipid-lowering therapy. Recent studies have found that the correct and continuous use of statins may reduce the risk of cardiogenic stroke recurrence, which is consistent with the findings of this study [58]. In clinical practice, AF patients often have various diseases, such as hypertension, diabetes, and coronary atherosclerotic heart disease, which create challenges for medical staff in the overall management of AF. In addition to focusing on the CHA_2_DS_2_-VASc score, factors such as blood lipid levels, smoking and drinking should be considered in AF patients, comprehensive health education should be provided, interventions for unhealthy lifestyles should be strengthened, and comprehensive management measures should be formulated to reduce the incidence rate and harm of IS. Wańkowicz P et al. [59] found that anticoagulant therapy alone cannot effectively prevent the occurrence of IS in NVAF patients. They believe that statins can be used for the secondary prevention of IS, and AF patients should improve their lifestyles.

### Study strengths and limitations

This study was the first to analyse the associations between LDL-C/HDL-C and IS in patients with NVAF. LDL-C/HDL-C combined with the CHA_2_DS_2_-VASc score had good discrimination and calibration in assessing the risk of IS in AF patients. This study was a multicentre case-control study with the following limitations. First, the study was a retrospective, non-longitudinal study. Only hospitalized patients were included; therefore, there was a selection bias. The control group and the case group did not match age, sex or other confounding variables that may affect stroke. Second, there are baseline differences between cases and controls, which may affect the results of the study. Finally, the study was performed in Xinjiang, China, and the findings cannot be generalized to the general population. A multicentre, prospective study will be considered in the future to further explore the relationship between LDL-C/HDL-C and IS in NVAF patients and to further study the relationships between LDL-C/HDL-C and measures in other related disciplines, such as proteomics and genomics.

## Conclusion

LDL-C/HDL-C > 1.22, smoking, BMI ≥24 kg/m^2^ and CHA_2_DS_2_-VASc score were independent risk factors for IS in NVAF patients, and LDL-C/HDL-C was the main risk factor. The size and severity of IS and its relation to NVAF and LDL-C/HDL-C should be considered in future studies. The discriminatory ability of the CHA_2_DS_2_-VASc score improved after LDL-C/HDL-C was added. AF patients have many comorbidities. In addition to basic anticoagulant therapy, attention should be paid to health education; the timely identification of controllable risk factors, such as dyslipidaemia and smoking; and improving patients’ lifestyle, strengthening self-management, reducing the risk of complications and improving quality of life.

## Data Availability

The datasets used and/or analysed during the current study are available from the corresponding author on reasonable request.

## Abbreviations

LDL-C/HDL-C: Low−/high-density lipoprotein cholesterol ratio
IS: Ischaemic stroke
NVAF: Non-valvular atrial fibrillation
AF: Atrial fibrillation
CT: Computed tomography
MRI: Magnetic resonance imaging
ECG: Electrocardiographs
TIA: Transient ischaemic attack
TG: Triglycerides
TC: Total cholesterol
Non-HDL-C: Non-high-density-lipoprotein cholesterol
PC: Principal component
VWF: Von Willebrand factor
OACs: Oral anticoagulants
BMI: Body mass index
